# Analysis of local genome rearrangement improves resolution of ancestral genomic maps in plants

**DOI:** 10.1186/s12864-020-6609-x

**Published:** 2020-04-16

**Authors:** Diego P. Rubert, Fábio V. Martinez, Jens Stoye, Daniel Doerr

**Affiliations:** 1 Faculdade de Computação – FACOM, Universidade Federal de Mato Grosso do Sul – UFMS, Campo Grande, Brazil; 2 Faculty of Technology and Center for Biotechnology (CeBiTec), Bielefeld University, Bielefeld, Germany

**Keywords:** comparative genomics, Ancestral genome reconstruction, Eudicot phylogeny, Local genome rearrangement

## Abstract

**Background:**

Computationally inferred ancestral genomes play an important role in many areas of genome research. We present an improved workflow for the reconstruction from highly diverged genomes such as those of plants.

**Results:**

Our work relies on an established workflow in the reconstruction of ancestral plants, but improves several steps of this process. Instead of using gene annotations for inferring the genome content of the ancestral sequence, we identify genomic markers through a process called *genome segmentation*. This enables us to reconstruct the ancestral genome from hundreds of thousands of markers rather than the tens of thousands of annotated genes. We also introduce the concept of *local genome rearrangement*, through which we refine syntenic blocks before they are used in the reconstruction of contiguous ancestral regions. With the enhanced workflow at hand, we reconstruct the ancestral genome of eudicots, a major sub-clade of flowering plants, using whole genome sequences of five modern plants.

**Conclusions:**

Our reconstructed genome is highly detailed, yet its layout agrees well with that reported in Badouin et al. (2017). Using local genome rearrangement, not only the marker-based, but also the gene-based reconstruction of the eudicot ancestor exhibited increased genome content, evidencing the power of this novel concept.

## Background

Ancestral genomes, that is, genome sequences of extinct species, are constituent for inferring phylogenies and for our understanding of evolutionary processes, such as adaptations to changing environmental conditions, the dynamics of genomes within populations and across species, and the study of pathogen-host interactions. At the same time, the study of ancestral sequences can give insights into gene function, regulatory networks, and molecular processes.

Flowering plants, with *eudicots* being their largest sub-clade, are an important subject of paleogenomic studies, not only because of their ecological significance and relevance for the crop industry, but also because the reconstruction of ancestral plant genomes is considered the most challenging endeavor of the field [1, 2]. The reconstruction of ancestral plant genomes is hard for multiple reasons: above all, plant genomes are often repetitive, as a result of one or more rounds of whole genome multiplication events that often occurred in their evolutionary past. Each round of polyploidization is followed by a period of dramatic genomic turnover in which the numbers of chromosomes and genes are reduced close to the order of magnitude prior to polyploidization. In doing so, chromosomes sustain large-scale rearrangements. Redundant genes and other functional units are randomly lost, leading to a fractionated layout of the genome when compared to its pre-polyploidization state. Furthermore, plant genomes are large, often exhibiting extensive intra- and inter-genic regions which themselves host repetitive elements such as transposons and long terminal repeats [3].

We present a workflow for inferring ancestral genome sequences with unlike higher degree of detail than obtained by currently available approaches. We further report on our ongoing progress in refining the resolution of the ancestral genome sequence of eudicots based on the genome sequences of five modern plants. We achieve the high degree of detail by improving several steps in the ancestral reconstruction process: First, our method identifies genomic markers, enabling the reconstruction of the ancestral genome from hundreds of thousands of markers rather than the tens of thousands of genes that have been annotated in the five eudicot genomes as of today. That way, our method does not need to rely on the quality of the gene annotation. But more importantly, our method can lead to a more comprehensive reconstruction of the ancestral genome content, as it is not restricted to those blocks of DNA attributed to protein-coding genes, and reveal new conserved blocks of yet unknown function. Second, it infers syntenic blocks across all extant genomes by tolerating inserted, deleted, and duplicated markers. Third, our method takes into account the internal structure of syntenic blocks for the reconstruction of contiguous ancestral regions by means of a local DCJ similarity measure, a novel measure proposed in this work.

### Eudicot evolution

**Fig. 1:**
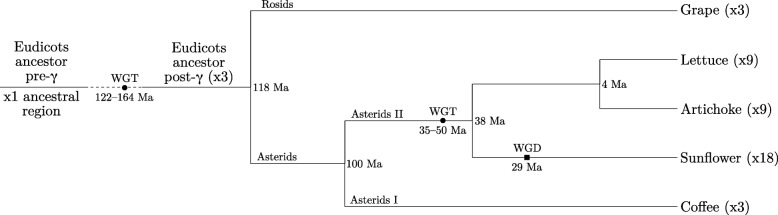
Eudicot phylogeny including grape and four asterids [4]. Circles and squares mark WGT and WGD events, respectively. Time is expressed in millions of years (Ma)

We study the eudicot phylogeny composed of grape, a representative of the rosids, and four asterids—artichoke, coffee, lettuce, and sunflower. Polyploidization is a major source of genomic innovation in plants and the studied eudicots are no exception to this rule [3, 4]: after the speciation of the eudicots and monocots around 140 to 150 millions years ago, the eudicot ancestor underwent a *whole genome triplication* (WGT), further denoted as *γ*, common to all known eudicots of today. Further polyploidizations occurred on subbranches, such as the WGT in the ancestor of the Asterids II group, to which sunflower, artichoke, and lettuce belong. The sunflower lineage underwent another *whole genome duplication* (WGD) event. Figure 1 gives an overview of the eudicot phylogeny and the described polyploidizations. The genome architecture of grape is closest to the post- *γ* ancestor, with only one chromosome fission and three chromosome fusions separating the two genomes. Therefore, in this work, the karyotypic architecture of the grape genome serves as proxy for reconstructing the genome of the post- *γ* ancestor.

### Related methods

The field of computational paleogenomics has established several methods to infer genome sequences of ancestral organisms from genome sequences of their extant descendants and relatives. In general, ancestral genome reconstruction is divided into two largely complementary—although interdependent—tasks: One is the inference of the genome’s *architecture*, i.e., the number, appearance, and composition of ancestral chromosomes. The other concerns the reconstruction of the *genome content* constituting the set of “building blocks” of the genome architecture that are the genomic markers, often represented by (protein coding) genes. Provided that the genomic coordinates of extant markers are known, the latter task coincides with the inference of homology classes, called *families*, and the determination of whether and how many members of each family are part of the ancestral genome content [5]. Most popular methods for reconstructing the ancestral genome architecture follow one of two strategies: either they make use of a *genome rearrangement model* to derive parsimonious rearrangement scenarios that explain the observed differences in modern genome architectures, or they infer syntenic blocks. These constitute conserved neighborhoods of individual pairs of markers, also denoted *adjacencies*, or neighborhoods of marker sets comprising more than two markers [6].

**Model-based reconstruction methods.** A prevalent genome rearrangement model is the *double-cut-and-join* (DCJ) model which defines rearrangement as an operation breaking the genome at two arbitrary positions and subsequently reconnecting the thus created four open ends in a new combination. In doing so, a break always occurs in the gap between two successive markers of the chromosomal sequence. For mostly all known rearrangement models, if duplications are not considered, the minimum number of operations to transform one given genome into another given genome can be computed efficiently. This number is also known as the distance between the given pair of genomes. Under the DCJ model, the distance is computed in linear time [7, 8]. However, considering one step further, the reconstruction of an ancestral genome for three given genomes under the DCJ model, also called the DCJ median problem, is already an NP-hard problem [9]. When duplications are taken into account, even pairwise distances between given genomes are NP-hard to compute for mostly all rearrangement models, including the DCJ distance [10].

Consequently, to deal with the aforementioned issues for the reconstruction of ancestral genomes, there are some proposed heuristic methods such as GASTS [11], MGRA [12] and Badger [13]. GASTS and MGRA operate under the command of parsimony, i.e., they aim to minimize the number of DCJ operations occurring along the edges of a given phylogeny. Conversely, Badger [13] considers a probabilistic model, using Bayesian analysis, aiming to solve the corresponding maximum likelihood problem. All methods assume that each marker is unique, with MGRA supporting that some markers may be missing in some of the genomes. As mentioned before, despite this unrealistic limitation (and at that unfounded in plant evolution), both objectives are computationally intractable, hence neither of the methods is exact but both implement fast heuristics that permit the analysis of biological datasets in practice.

**Synteny-based reconstruction methods.***Syntenic blocks* are blocks of two or more extant genome sequences that are *homologous*, i.e., they originate from the same block of a common ancestral sequence. Methods that make use of inferred syntenic blocks must resolve conflicts between contradicting neighborship relations of genomic markers imposed by these blocks in order to derive a total or partial, sequential or circular order of common ancestral markers. The most popular such method, ANGES [14], identifies a subset of neighborship relations that can be displayed by a PQ-tree, a data structure for capturing local variations in a set of permutations. ANGES’ procedure implies that each family can contribute at most one marker to the ancestral genome content. This severely limits the applicability of the method for the reconstruction of ancestral plant genomes, where multiple rounds of polyploidy have frequently occurred, resulting in multiple copies of each gene. Alternative methods such as PMAG [15] and DeCoStar [16] use likelihood estimation to infer ancestral gene orders, yet are limited to process adjacencies only. Nevertheless, DeCoStar infers evolutionary trees of marker adjacencies and therefore can handle evolutionary events such as duplication, insertion, and loss [6].

Independent of the strategy, the outcome of both approaches are *contiguous ancestral regions* (CARs), that detail the composition of ancestral chromosomes (or parts thereof) as well as the relative order of their contained genomic markers. Such order may not be entirely fixed—the resolution of ancestral marker orders depends on the input data [17], the method of choice [6], and its alacrity to proclaim neighborship relations derived from the analysis of extant genomes as ancestral. Many methods output multiple candidates for ancestral gene order, either because their strategy is based on sampling, or because it is subject to optimization criteria that give rise to many co-optimal solutions.

In an attempt to combine the two complementary strategies, we developed a rearrangement-aware synteny-based reconstruction method that extends an established pipeline for ancestral genome reconstruction in plants [3, 5] used in multiple studies [4, 18, 19]. Our method refines the genome content of syntenic blocks prior to deriving contiguous ancestral sequences. To this end, we introduce a concept analogous to *local sequence alignment* that we call “local genome rearrangement”.

The remainder of this paper is organized as follows: In the next section, we introduce basic concepts and notation that will be used in the description of our ancestral reconstruction pipeline in section “Methods”. Subsequently, in section “Results” we provide a comprehensive report of our reconstruction of the eudicot ancestor. Finally, in section “Conclusions” we review our results in relation those of Badouin et al. [4].

## Preliminaries

### Genomic sequences

A (genomic) marker is a block of DNA sequence represented by the unique identifier of its associated *family*. The double-strandedness of the DNA imposes a relative orientation to each marker *g*: If *g*’s orientation conforms with the (predetermined) reading direction of its sequence *S*, *g* is denoted in *S* by itself. Otherwise, it has reverse orientation and is denoted in *S* by −*g*. If the orientation of a marker *g* is irrelevant, we denote by |*g*| the marker itself, omitting its orientation. A *genome* is a collection of marker sequences, also called *chromosomes*.

Given a sequence *S* over *n*=:|*S*| markers, *S*[*i*] denotes the marker at the position *i* and $\mathcal {G}(S) := \bigcup _{i=1}^{n} \{|S[i]|\}$ is the *(genome) content* of *S*. Further, we define the *multiplicity* of a marker *g* in sequence *S* as *m*_*S*_(*g*):=|{*i* ∣1≤*i*≤*n* and |*S*[*i*]|=*g*}|. A sequence *S* is *duplicated* if any of its markers has multiplicity larger than one. Such markers are *duplicate* markers. Further, two sequences *S* and *T* are *balanced* if $\mathcal G(S) = \mathcal G(T) =: \mathcal G$ and each marker $g \in \mathcal G$ has the same multiplicity in both genomes, i.e., *m*_*S*_(*g*)=*m*_*T*_(*g*). The concepts of multiplicity, duplication, and balance naturally propagate to collections of marker sequences and thus apply equally to genomes.

The *interval* [*i*,*j*] in sequence *S* gives rise to the substring *S*[*i*,*j*]=*S*[*i*]*S*[*i*+1]⋯*S*[*j*], with 1≤*i*≤*j*≤|*S*|. An interval [*i*,*j*] of sequence *S* is called *maximal* if it cannot be extended to its left or right without changing the genome content, i.e., either *i*=1 or $\mathcal {G}(S[i-1,j]) \neq \mathcal {G}(S[i,j])$ and either *j*=*n* or $\mathcal {G}(S[i,j+1]) \neq \mathcal {G}(S[i,j])$. Given two sequences *S* and *T*, a pair of intervals [*i*,*j*] of *S* and [*k*,*l*] are *common intervals* if $\mathcal G(S[i,j]) = \mathcal G(T[k,l])$. A sequence *T* is a *subsequence* of *S* if *T*=*S*[*i*_1_] *S*[*i*_2_]⋯*S*[*i*_*k*_] such that 1≤*i*_1_<*i*_2_<⋯<*i*_*k*_≤|*S*|.

### DCJ model

A non-duplicated genome *G* can be represented by its set of *adjacencies*, where each marker *g* of its chromosomes is represented by a pair of its head and tail extremity *g*^h^ and *g*^t^, respectively, i.e., by pair (*g*^t^,*g*^h^) if marker *g* lies in reading direction of the chromosome, otherwise by (*g*^h^,*g*^t^). Then the set of adjacencies $\mathcal A(G)$ of genome *G* with *n* markers is given by the set of incident extremities of consecutive markers, where the first and last adjacencies of linear chromosomes correspond to the outermost extremities of the first and last markers, called *telomeric* adjacencies. A genome *G* evolves by a DCJ operation that splits any two adjacencies into their four extremities (where telomeric adjacencies are split into the single constituting extremity and an empty extremity) and recombines them into two new adjacencies.

Given two balanced genomes *G* and *H*, the minimum number of DCJ operations required to transform *G* into *H* is the *DCJ distance* between *G* and *H*, denoted by d_DCJ_(*G*,*H*). It is a classic result of the field that the DCJ distance between non-duplicated balanced genomes *G* and *H* over *n*=|*G*|=|*H*| markers can be computed in linear time by counting the number of (even) cycles *c* and odd paths *o* in the *adjacency graph* *AG*(*G*,*H*) [7, 8]. The adjacency graph *AG*(*G*,*H*) is a bipartite multigraph (*U*,*V*,*E*), with vertex sets $U = \mathcal A(G)$ and $V = \mathcal A(H)$ and edge multiset *E*={(*u*,*v*) *with multiplicity* |*u*∩*v*|:*u*∈*U*,*v*∈*V* *and* *u*∩*v*≠*∅*}. Then the DCJ distance between *G* and *H* is given by d_DCJ_(*G*,*H*)=*n*−*c*−*o*/2. However, the calculation of the DCJ distance for general balanced genomes is NP-hard [10].

## Methods

We present ANGORA (ANcestral reconstruction by local GenOme Rearrangement Analysis), a workflow for the reconstruction of ancestral plant genomes. Our method is based on previous work by Salse [3, 5], but additionally includes a preceding step to identify genomic markers. Subsequently, syntenic blocks are identified and finally used to derive contiguous ancestral regions.

### Identification of genomic markers

**Fig. 2:**
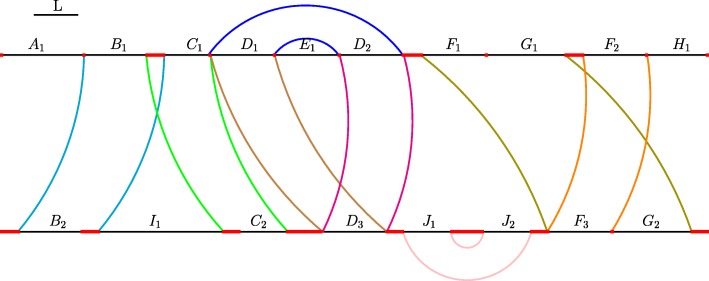
Example of a segmentation of two DNA sequences. The minimum segment length *L* is indicated by the line in the upper left corner, waste regions are marked by thick red lines, pairwise alignment boundaries by colored arcs. Capital letters above atoms indicate their family membership

We obtain genomic markers by solving the genome segmentation problem [20]. Informally, the objective of genome segmentation is the decomposition of a DNA sequence into families of non-overlapping segments, called *atoms*. To this end, genome segmentation takes as input pairwise alignments of the DNA sequence onto itself and requires that no alignment boundary lies within any of the created atoms. Note that the genome segmentation problem for multiple DNA sequences is simply defined as the segmentation problem of the concatenated DNA sequences. A trivial segmentation would establish every single character of the input sequence into an atom of its own, thus satisfying the stated criteria. To avoid such meaningless segmentation, a minimal length requirement is imposed on the constructed atoms. Any nucleotide that is not covered by an atom resides in a *waste region*. Figure 2 shows an example of a segmentation of two DNA sequences. The objective of the *genome segmentation problem* (GSP) is the construction of a segmentation that minimizes the total number of nucleotides located in waste regions. In 2013, Visnovská and colleagues have proven its intractibility and devised a heuristic called IMP for its solution [21].

### Discovery of syntenic blocks

The identification of syntenic blocks in highly diverged genomes, such as the five eudicots subject to our study, is challenging. That is because on the one side, the notion of synteny is highly flexible, simultaneously allowing an entire chromosome to be classified into a single syntenic block, as well as individual segments thereof [22]. On the other side, multiple rounds of mutations such as insertions, deletions, duplications, and rearrangements can scramble and decompose syntenic blocks into barely recognizable units. Methods to identify syntenic blocks under such conditions must be equally flexible: they must tolerate comprehensive changes in the order and multiplicity of genomic markers, but at the same time pick up the signal of synteny on all levels of granularity, ranging from chromosome level down to synteny of individual pairs of genomic markers.

One such method that is particularly fast (speed is another important concern of this step in the ancestral reconstruction workflow) is Gecko3 [23], which identifies syntenic blocks by discovering approximate common intervals in marker sequences. These are sets of intervals with associated genome content $\mathcal G$ such that the symmetric difference between each interval and $\mathcal G$ is bounded by *δ*^sum^ and, more specifically, the number of excessive (i.e., *inserted*) markers is bounded by *δ*^add^, and the number of missing markers by *δ*^loss^. Gecko identifies the genome content of a set of intervals by a referenced-based approach. In doing so, a designated genome (the “reference”) is taken as scaffold for the discovery of approximate common intervals in the other genomes. Any interval in the reference defines the genome content $\mathcal G$ of an interval set. Gecko3 can find approximate common intervals with multiple occurrences within a single sequence and also provides a *quorum* parameter *q* by which approximate common intervals can be discovered that are conserved only in a subset of genomes of size at least *q*.

### Family refinement using local dCJ similarity

Similar to local sequence alignment, local genome rearrangement aims at identifying highly conserved pairs of substrings of two given marker sequences. For the same reason that the edit distance cannot be used for computing local alignments, the DCJ distance cannot be used to compute local rearrangements: Both would favor pairs of substrings that minimize the number of edit operations independent of their length, thereby giving pairs of small substrings—in particular the pair of empty strings—a dishonest advantage. Clearly, the method of choice are similarity measures that, rather than solely penalizing *dissimilarity*, quantify *similarity*. Conversely, global measures of DCJ similarity, such as those proposed by some of us [24, 25] that only maximize the (weighted) number of cycles and paths in the adjacency graph, are unsuitable as well: In search of locally similar sequences, it is not sufficient to reward only similarity (then, a best local solution would always correspond to a global solution), but it is necessary to also penalize dissimilarity.

With our goal of studying highly diverged genomes, we designed a procedure able to tolerate all kinds of differences caused by insertion, deletion, or duplication of one or several genomic markers. To this end, we first discover referenced-based approximate common intervals in the studied genomes. Each discovered set of intervals gives rise to a set of pairs of substrings between the reference and the remaining genomes for which local rearrangement scores are calculated.

**Fig. 3:**
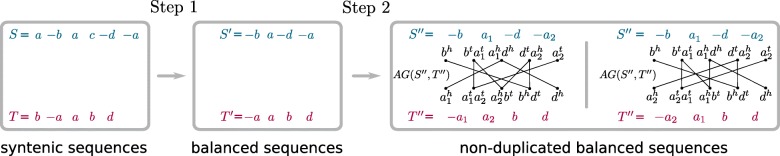
Illustration of the procedure for computing local DCJ similarity scores from a pair of syntenic marker sequences

Let *S* and *T* be two substrings associated with one of these pairs of approximate common intervals. Our method proceeds in two steps that are illustrated by an example in Fig. 3. First, pairs of sequences *S*^′^,*T*^′^ are identified such that (i) *S*^′^ is a subsequence of *S*, and *T*^′^ of *T*, (ii) *S*^′^ and *T*^′^ are balanced, and (iii) for each marker $g \in \mathcal G(S')$ holds true that $\phantom {\dot {i}\!}m_{S^{'}}(g) = \min (m_{S}(g), m_{T}(g))$. The last constraint ensures maximality of the balanced subsequences.

Sequences *S*^′^ and *T*^′^ are then subjected to a second procedure that finds one-to-one assignments between all markers of the two sequences, thus further refining them to non-duplicated balanced sequences *S*^′′^ and *T*^′′^. Eventually, those pairs of balanced sequences *S*^′′^ and *T*^′′^ are identified that maximize the following formula:

$${\begin{aligned} {{s_{\textsc{DCJ}}}}\left(S^{\prime\prime}, T^{\prime\prime}\right) = \sum_{C \in \mathcal{C}}{f(|C|)} + \frac{1}{2} \left (\sum_{O \in \mathcal{O}}{f(|O|+1)} + \sum_{E \in \mathcal{E}}{f(|E|+2)}\right) - d \cdot p\:, \end{aligned}} $$

where $\mathcal {C}, \mathcal {O}$ and $\mathcal {E}$ are the sets of cycles, odd paths, and even paths in the constructed adjacency graph of *S*^′′^ and *T*^′′^,*d*:=|*S*|+|*T*|−(|*S*^′′^|+|*T*^′′^|) is the number of deleted markers and *p* is the deletion penalty. Function $f : 2\mathbb {N} \rightarrow \mathbb {R}$ scores each cycle and path proportional to its length—where, as in earlier literature [26, 27], the lengths of paths are corrected so that structures with the same sorting distance have the same “length”. Because short cycles and paths are indicators of similarity, whereas long cycles and paths suggest the opposite, we found a simple realization of *f* that has been working well in our experiments:

1 $$ f(l) = \frac{2 - l}{L - 2} + 1\:.  $$

Our function *f* makes use of a constant *L*, a length threshold that demarcates short from long cycles and paths.

**Fig. 4:**
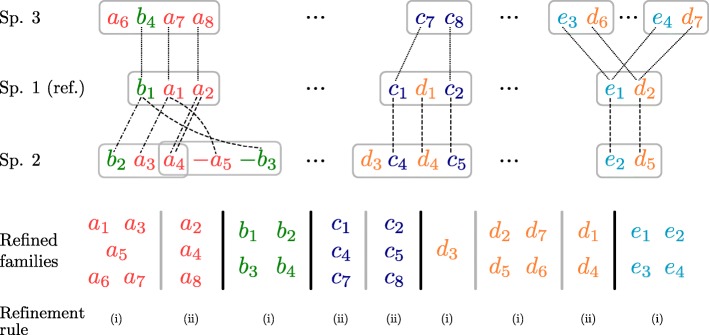
Example of family refinement for hypothetical species 1 (reference), 2 and 3. For each species, occurrences of exemplary syntenic blocks are shown, indicated by gray boxes. Families *a*, *b*, *c*, *d* and *e* are refined based on one-to-one assignments (indicated by dotted, dashed, and dotted-dashed lines) of local DCJ similarity calculations between the reference and the other two sequences. Subscript indices are used to distinguish markers of the same family

In reconstructing ancestral genomes, we use non-duplicated balanced sequences *S*^′′^ and *T*^′′^ identified by the described optimization procedure to refine the genomic marker families across the entire genomic dataset. This enables substantial improvement in determining the ancestral genome content, as detailed in the next section. To this end, we implemented a procedure that takes unambiguous one-to-one assignments across overlapping syntenic blocks to decompose their marker families into disjoint subsets. Further, if non-overlapping sets of syntenic blocks share markers from the same family, this family is also decomposed into disjoint subsets corresponding to the syntenic block affiliation of its members. The refinement process is depicted in Fig. 4 and described here in detail.

Two sets of syntenic blocks *B*={*b*_1_,*b*_2_,…} and *B*^′^={*b*1′,*b*2′,…}*overlap* if any block of *B* shares a genomic marker with a block of *B*^′^. Given a collection $\mathcal B$ of sets of syntenic blocks, we define a graph *G*=(*V*,*E*) with vertex set $V = \mathcal B$ and edge set $E = \{(B,B') \mid \{B, B'\} \subseteq \mathcal B: B \text { and } B' \text { overlap}\}$. Each connected component of *G* induces a (maximal) *overlapping set* of sets of syntenic blocks. For each such overlapping set *O*, new (sub-) families are created according to the following two rules:

1. Let *F* be a family of markers and *F*_*O*_⊂*F* be the subset of markers embedded in any set of syntenic blocks of *O*. For each such family *F* for which *F*_*O*_≠*∅*, a new family *F*_*O*_ is created.
2. Let *m*_1_∈*F*_*O*_ be a marker of reference sequence *S*_1_ and let $S_{2}, \dotsc, S_{k}$ be the *k*−1 genomic sequences other than the reference such that each *S*_*i*_,2≤*i*≤*k*, has a marker assigned to *m*_1_ in at least one local DCJ similarity computation. If, for all 2≤*i*≤*k*, *m*_1_ is assigned to the same marker *m*_*i*_ of *S*_*i*_ in every local DCJ similarity computation between the reference and *S*_*i*_, then the set of markers $\{m_{1}, m_{2}, \dotsc, m_{k}\}$ induces a new family. This rule further refines new families created by rule (i).

### Reconstruction of cARs

The last step of the workflow is conducted with ANGES and is the same as in the original workflow of Salse [3]. ANGES takes as input syntenic blocks or identifies them by discovering maximal common intervals (or constrained variants thereof). The identified intervals are then either weighted by user-provided data, or according to the occurrences in the extant genomes and subsequently used to construct and output a PQ-tree. A *PQ-tree* is a hierarchical data structure capable for the lossy representation of all common intervals of two or more permutations. To this end, PQ-trees make use of two kinds of nodes: *P*-nodes, which do not impose any order of their child nodes, and *Q*-nodes, which indicate a linear order of their children.

## Results

### Genome segmentation

**Fig. 5:**
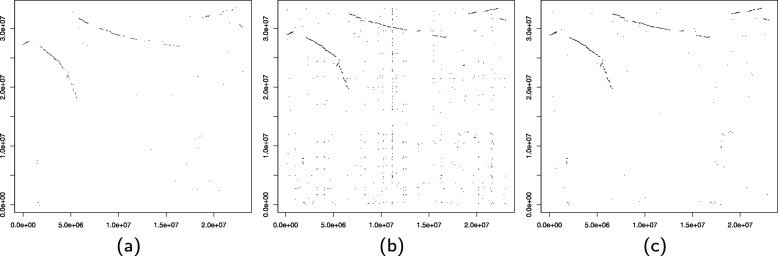
Dot-plot for chromosomes 1 of grape and 11 of coffee given by (**a**) CoGe [29], (**b**) our computed LASTZ alignments, and (**c**) after genome segmentation.

To enable the processing of large genomic datasets such as the one at hand, we have re-implemented the heuristic IMP described in [21] in C++ and adapted it for parallel computation. Our software, named GEESE (GEnomE SEgmentation), is included in the ANGORA workflow, but can also be obtained separately. (For details, see Section “Availability of data and materials” below.) Following the approach described in [21], we used LASTZ [28] to compute local sequence alignments between all pairs of genomic sequences from the five eudicots. In doing so, we chose alignment parameters (see Supplementary Material) that improved the clarity and detail of dot-plots of inter-species chromosome pairs, such as those shown in Fig. 5. We further compared our dot-plots with those generated by CoGe [29], a popular platform for comparative genomics analyses, under default parameter settings. Based on the DAGChainer [30] algorithm, CoGe provides functionality to identify genomic markers in pairs of genomes. Despite CoGe’s method for identifying genomic markers being unrelated to ours, the dot-plots are similar, suggesting that the constructed genome segmentation is robust and unbiased.

Based on 246 million pairwise local alignments reported by LASTZ, IMP derived 640 thousand atoms of minimum length 100bp which are associated with families occurring in two or more genomes. In comparison, the total amount of annotated genes in the five eudicots is around 140 thousand [4]. Table 1 shows for the same five eudicots the number of genes that have been used in ancestral reconstruction by Badouin *et al.* [4] and information on the annotated genes and genomic markers obtained from latest genome databases. We subsequently removed those markers/genes from their genomic sequences that were associated to families not shared by at least two genomes. That way we obtained 9,374 families from the set of annotated genes, with average size 6.5 and occurring in 4.1 genomes on average. For genomic markers, 123,218 families were derived, with average size 5.7 and occurring in 2.9 genomes on average.

**Table 1:** Genes, markers and families in each of the five eudicots

	Badouin et al.	Annotated genes				Genomic markers			
	total genes	total genes	shared genes	families	avg. fam. occ.	total markers	shared markers	families	avg. fam. occ.
Grape	26,346	23,180	10,514	7,675	1.4	145,152	50,103	31,533	1.6
Coffee	25,574	21,971	13,267	9,374	1.4	97,735	34,125	23,598	1.4
Artichoke	27,121	23,394	11,124	7,034	1.6	396,323	153,448	92,401	1.7
Lettuce	12,841	37,829	11,249	7,032	1.6	860,023	178,217	83,806	2.1
Sunflower	52,243	58,022	14,604	7,300	2.0	1,364,948	223,500	93,526	2.4

For each species, shared genes (markers) represents the amount of genes (markers) occurring in at least one other genome. Average family occurrences shows, for families occurring in at least two genomes, how many times each family occur on average in each genome

### Syntenic blocks

We extended Gecko3 by our method for computing local DCJ similarity scores. We have used in Gecko3 a *default* and a *relaxed* table (see Table 4 in the Supplementary Material) to set indel thresholds depending on the size of the shared genome content of compared intervals. Using grape as reference genome, we ran Gecko3 with varying quorum, and default and relaxed indel thresholds. A list of results for each of those parameter settings is shown in Table 2. For the calculation of the local DCJ similarity scores of reported syntenic blocks, we set the deletion cost to *p*=0.25 and the length threshold of function *f* (see Eq. (1)) to *L*=8. Gecko3 reported 48,877 syntenic blocks for our final choice of parameter settings (see Table 2, run 2). Each such block occurred on average 1.0, 1.1, 1.6, 1.7, and 2.1 times in grape, coffee, artichoke, lettuce and sunflower, respectively. These values are compatible with the ancestral polyploidization events of their phylogeny.

**Table 2:**
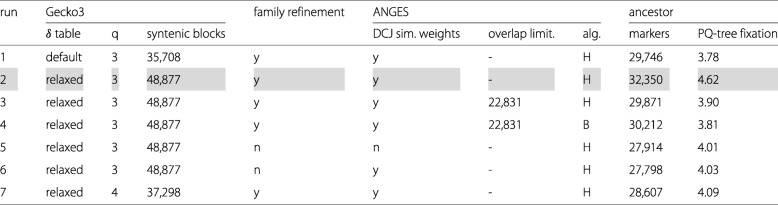
Overview of ancestral reconstructions under varying parameters of our pipeline

Our final choice of parameters is highlighted in gray

### Contiguous ancestral regions

**Fig. 6:**
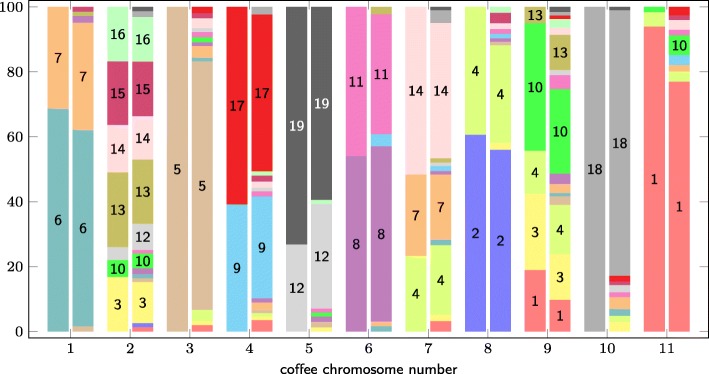
Shared content between coffee and grape genomes in the reconstructed ancestor. For each coffee chromosome (x-axis), each pair of bars shows the proportion shared with grape chromosomes (indicated by the color and chromosome number inside each bar segment) by the ancestral genome of Badouin et al. [4] (left) and ours (right), respectively. For better visualization, proportions of ancestral genome contents below 1% are not shown

Our reconstructed genome of the eudicot ancestor is composed of 32,788 markers distributed across 3,153 CARs, with the largest CARs comprising between 50 and 100 markers. This ancestral genome is in remarkably high agreement with that constructed by Badouin and colleagues [4], despite the fact that quite different sets of genomic markers have been used: By comparing the proportions of genomic markers attributed to each ancestral chromosome with the proportions derived from Badouin et al.’s gene-based reconstruction, the two ancestral genomes differ only on average in 3.2%, with standard deviation of 3.7%. Figure 6 shows the comparison of ancestral genome content w.r.t. coffee and grape chromosomes of this analysis.

We investigated whether our family refinement approach using local genome rearrangement improved the ancestral reconstruction. We followed three different paths: First, we quantified the impact that the family refinement procedure has on the ancestral genome content. Second, to untangle the effects of this refinement procedure from marker-based vs. gene-based reconstruction, we re-ran our reconstruction pipeline, this time using the latest gene annotations of the five eudicot genomes. To this end, we constructed gene families as described in [3] by binning genes with *cumulative identify percentage* (CIP) of 60% and *cumulative alignment length percentage* (CALP) of 70% [31]. Third, we quantified the *fixation* in ancestral marker order by measuring the average number of children of *Q* nodes in the PQ-tree constructed by ANGES.

The results obtained with these modified workflows make us believe that the family refinement indeed has a non-negligible positive effect: First, when skipping the local rearrangement-based family refinement procedure, the number of markers in the reconstructed ancestor amounted to 27,798. In other words, the family refinement led to an increase of 18% in ancestral genome content. Second, in the gene-based reconstruction, we observed similar results: Whereas local rearrangement-based family refinement led to 6,961 ancestral genes, without refinement their number decreased to 5,945. Third, the average number of children of *Q* nodes increased through rearrangement-based family refinement from 4.0 to 4.6 in marker-based reconstruction. Again, we observed the same trend in the gene-based reconstruction (4.2 without and 5.0 with refinement).

In addition, we studied the parameter space of our pipeline by conducting multiple runs listed in Table 2. By far the biggest impact w.r.t. the size of the ancestral genome content had the parameter settings of Gecko3, i.e., the choice of *δ* table, and the quorum parameter *q* (cf. runs 1, 2, and 7). ANGES weights syntenic blocks to guide the choice of discarding some of them in cases of conflict. We provided our local DCJ similarity scores as weights, but also ran ANGES on its internally computed weights, observing only minor differences, although surprisingly in favor of ANGES’ weights (cf. runs 5 and 6). Furthermore, ANGES provides two different algorithms for reconstructing the PQ-tree: a heuristic (H) and a branch-and-bound (B) algorithm. Although the latter can recruit more markers into the ancestral genome content (cf. runs 3 and 4), it has a much higher running time, that only allowed us to compute ancestral reconstructions when we dramatically reduced the number of provided syntenic blocks. We limited then the number of overlapping syntenic blocks to 30 and chose (heuristically) promising subsets whenever this limit was exceeded. The number of syntenic blocks after this filtering step dropped to 22,831, reducing the number of markers and the fixation of the ancestral PQ-tree.

## Conclusions

Recently, Badouin and colleagues reconstructed the eudicot ancestor from the gene annotations of grape, coffee, artichoke, lettuce and sunflower and arrived at an ancestral genome comprising 6,525 genes [4]. In this work, we followed the same workflow for ancestral reconstruction, but made multiple improvements: First, instead of using annotated genes, we identify genomic markers and use them as building blocks of the ancestral sequence, allowing us to reconstruct both intra- and intergenic blocks of DNA. Second, instead of using CloseUp [32], a statistical method for discovering syntenic blocks in pairs of genomic sequences, we use Gecko3 [23], which computes exact solutions under a principled definition of synteny [22, 33] in multiple sequences. Third, based on the concept of local genome rearrangement introduced in this work, we score syntenic blocks and refine the family assignment of their contained genomic markers. Our improvements lead to a reconstruction of the ancestral eudicot genome that is composed of 32,788 markers distributed across 3,153 CARs. Remarkably, the layout of our ancestral genome differs on average only in 3.2% from that Badouin et al. [4]. Our method is also applicable to gene-based reconstruction, where it increased the genome content of the eudicot ancestor to 6,961 reconstructed genes while differing on average only in 4.6*%* from Badouin et al.’s reconstruction.

## Supplementary information

**Additional file 1** This PDF document describes the datasets used in the main text, where to obtain these datasets, how they were prepared to be used in the workflow, and the parameters for the integrated tools used in the research.

## Data Availability

GEESE is available at https://gitlab.ub.uni-bielefeld.de/gi/geese. Gecko3-DCJ is available at https://gitlab.ub.uni-bielefeld.de/gi/gecko-dcj. ANGORA is available at https://gitlab.ub.uni-bielefeld.de/gi/angora. Experimental data is available at http://doi.org/10.4119/unibi/2936848.

## Abbreviations

CALP: Cumulative alignment length percentage
CAR: contiguous ancestral regions
CIP: Cumulative identify percentage
DCJ: Double-cut-and-join
DNA: Deoxyribonucleic acid
GSP: Genome segmentation problem
Ma: Millions of years
WGD: Whole genome duplication
WGT: Whole genome triplication
