# Supplementary material for: Analysis of local genome rearrangement improves resolution of ancestral genomic maps in plants
Source: BMC Genomics. 2020 Apr 16;21(Suppl 2):273. doi: 10.1186/s12864-020-6609-x (PMC7160886; doi:10.1186/s12864-020-6609-x)
Supplement: Supplementary file 1 — Additional file 1 This PDF document describes the datasets used in the main text, where to obtain these datasets, how they were prepared to be used in the workflow, and the parameters for the integrated tools used in the research. [file 12864_2020_6609_MOESM1_ESM.pdf]

Analysis of local genome rearrangement improves resolution of ancestral genomic maps in plants

Diego P Rubert, Fábio H V Martinez, Jens Stoye and Daniel Doerr

Supplementary Material

This supplementary material describes datasets used in the main text of this publication, including where they can be obtained and how they were prepared to be used with our workflow. Further, the parameters for the integrated tools that are used in this study are described. Data used by and generated in this study are available at `http://doi.org/10.4119/unibi/2936848`.

Contents

1 Genome databases . . . 1

2 Workflow availability . . . 2

3 LASTZ: local sequence alignment . . . 2

4 GEESE: genome segmentation . . . 3

5 Filtering families . . . 3

6 Gecko3-DCJ: discovering syntenic blocks . . . 3

7 ANGES: Ancestral genome reconstruction . . . 4

8 Calculation of proportions of shared content between genomes . . . 5

1 Genome databases

Following are the genome assemblies used in this study and where they can be obtained:

Grape *Vitis vinifera* (wine grape), assembly GCA_000003745.2 12X, NCBI,
`https://www.ncbi.nlm.nih.gov/genome/401?genome_assembly_id=214125`

Coffee *Coffea canephora*, assembly v1.0, Coffee Genome Hub,
`http://coffee-genome.org/coffeacanephora`

Artichoke Cynara cardunculus var. scolymus, assembly GCA_001531365.1 CcrdV1, NCBI,
`https://www.ncbi.nlm.nih.gov/genome/11286?genome_assembly_id=372115`

Lettuce *Lactuca sativa* var Salinas, assembly L. sativa cv Salinas V8, Lettuce Genome Resource,
`http://lgr.genomecenter.ucdavis.edu/Private/Downloads/BulkDownload.php`

Sunflower *Helianthus annuus* (common sunflower), assembly GCA_002127325.1 HanXRQr1.0, NCBI,
`https://www.ncbi.nlm.nih.gov/genome/351?genome_assembly_id=317475`

Data preparation. Our workflow takes as input genome data in the form of GenBank files. However, the genome data obtained from different databases (e.g. JGI, NCBI, Ensembl) are in different formats. Conversion of these formats was necessary to standardize the dataset. Further, scaffolds not associated to any chromosome but which were also present in the data files have been filtered out. In detail, we have:

Sunflower: Removed scaffolds and added `locus_tag` to all `CDS` entries.

Grape: Removed scaffolds and added `locus_tag` to all `CDS` entries.

Artichoke: Removed scaffolds and added `locus_tag` to all `CDS` entries.

Coffee: A GenBank file was built from Fasta and GFF files by using the auxiliary script `fa+gff2gbk.py` included in the workflow under the `data/scripts` folder. 12,996 unmapped scaffolds (totaling 204 Mb) were removed.

Lettuce: A GenBank file was built from Fasta and GFF files by using the auxiliary script `fa+gff2gbk.py` included in the workflow under the `data/scripts` folder. Chromosome names had to be shortened, since they were too long and were causing errors in BioPython's GenBank writer.

2 Workflow availability

The workflow implementation, named ANGORA, is publicly available at `https://gitlab.ub.uni-bielefeld.de/gi/angora`. All steps necessary to download and configure the workflow, its dependencies, and how it can be run are described in the provided `README.md`. The workflow package includes two small sample datasets, one having 3 unichromosomal genomes of simulated species, and one having 3 multichromosomal genomes of real species (*Ostreococcus* green algae).

3 LASTZ: local sequence alignment

The tool used for aligning DNA sequences is LASTZ, which can be obtained at `https://github.com/lastz/lastz`. However, LASTZ has a bug that, depending on the parameters choice, outputs inconsistent data. By the time this study was published, there was no public release correcting the bug. Therefore, our workflow contains a custom LASTZ hotfix version that can be found at `https://gitlab.ub.uni-bielefeld.de/gi/lastz-hotfix`. The complete list of LASTZ parameters can be found at `https://lastz.github.io/lastz/`. Parameters and their settings used in our eudicot study are:

```
--notransition
--step=10
--gapped
--hspthresh=6000
--nochain
--gfextend
--ambiguous=iupac
--masking=5
--filter=identity:70
```

Values under 6000 for `hspthresh` resulted in excessive noise for the eudicots dataset. Table 1 shows how to configure LASTZ parameters in the `config.yaml` file of our workflow.

Table 1: Mapping of parameters for sequence alignment.

LASTZ parameter	→	Workflow configuration (`config.yaml`) entry
any parameter `--parameter`		`lastz_params: --parameter ...`
any parameter `--parameter=<value>`		`lastz_params: --parameter=<value> ...`

4 GEESE: genome segmentation

For genome segmentation we have used GEESE, an efficient parallel implementation of the IMP algorithm [2] written in C++, which can be found at https://gitlab.ub.uni-bielefeld.de/gi/geese. The parameters used are:

- Minimum atom length (--minLength): 100;
- Minimum percent identity (--minIdent): 30;
- Minimum alignment block size (--minAlnLength): 13;
- Maximum gap length inside of an alignment (--maxGap): 100.

Table 2 shows how to configure GEESE parameters in the config.yaml file of our workflow.

Table 2: Mapping of parameters for segmentation.

GEESE parameter	→	Workflow configuration (config.yaml) entry
--minLength <value>		marker_min_length: <value>
--minIdent <value>		sgmtn_alignment_ident: <value>
--minAlnLength <value>		sgmtn_alignment_minlen: <value>
--maxGap <value>		sgmtn_alignment_maxgap: <value>

5 Filtering families

After the genome segmentation, the resulting families pass through a filtering step made by the script that post-processes the genome segmentation output (atoms2cog.py). In this filtering step, families that are too large or occur only once can be removed. We have filtered out families according to the following rules:

- Families larger than 98% of all the families (--percent 98);
- Families that have a single representative (--ignore0).

Table 3 shows how to configure family filtering parameters in the config.yaml file of our workflow.

Table 3: Mapping of parameters for family filtering.

atoms2cog.py parameter	→	Workflow configuration (config.yaml) entry
any parameter --parameter		cog_params: --parameter ...
any parameter --parameter <value>		cog_params: --parameter <value> ...

6 Gecko3-DCJ: discovering syntenic blocks

Syntenic blocks in our workflow are found by Gecko3-DCJ (https://gitlab.ub.uni-bielefeld.de/gi/gecko-dcj), which finds (referenced-based) approximate common intervals and quantifies their structural similarity by means of the local DCJ similarity score. A collection of intervals associated with genome content $\mathcal{G}$ is approximate common if the symmetric difference between the genome content of each interval and $\mathcal{G}$ is bounded by δ^{sum} and, more specifically, the number of excessive (i.e., *inserted*) markers is bounded by δ^{add}, and the number of missing markers by δ^{loss}. The two δ tables (default and relaxed) used in the eudicots study are shown in Table 4 (-dT <table> parameter). The quorum parameter q was set to 3 using the -q 3 option. All Gecko3-DCJ options used in command line can also be set using its graphical interface.

The local DCJ similarity formula as defined in the main text requires a function $f : 2\mathbb{N} \to \mathbb{R}$ that scores each cycle and path proportional to its length. In this study we have used the function

$$f(l) = \frac{2-l}{L-2} + 1\,, \tag{1}$$

Table 4: δ tables used by Gecko3.

Default				Relaxed			
Size	δ^{add}	δ^{loss}	δ^{sum}	Size	δ^{add}	δ^{loss}	δ^{sum}
2	0	0	0	2	0	0	0
3	0	0	0	3	1	0	1
4	1	0	1	4	1	1	1
5	2	1	2	5	2	1	2
6	3	2	3	6	3	2	3
7	4	2	4	7	4	2	4
8	5	3	5	8	6	3	7
9	6	3	6	9	8	4	8

where l is the length of the cycle or path and L is a length threshold that demarcates short from long cycles and paths (called *borderline cycle length* in Gecko3-DCJ). In the calculation of the local DCJ similarity, enabled in Gecko3-DCJ by the `--dcj` option, the value $L = 8$ was used (`--dcjBorderline 8`). See Figure 1 for an example of the function f for $L = 8$. We have also allowed the use of heuristics to compute the local DCJ similarity when gene families are too large (`--dcjUseHeuristics`). As for the deletion cost d, we have used the value 0.25 (`--dcjPenality 0.25`).

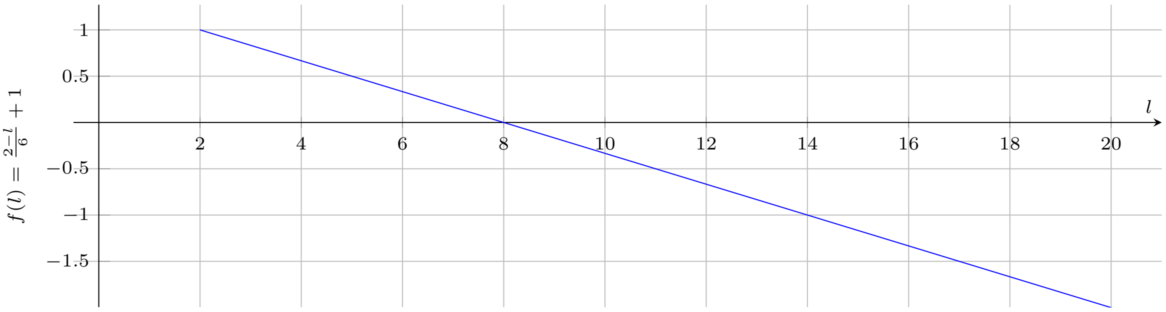

Figure 1: Plot of function f as defined in Equation 1 for $L = 8$. The horizontal axis represents the cycle or path length l and the vertical axis represents the score $f(l)$.

Table 5 shows how to configure Gecko3-DCJ parameters in the `config.yaml` file of our workflow.

Table 5: Mapping of parameters for discovery of syntenic blocks.

Gecko3-DCJ parameter	→	Workflow configuration (`config.yaml`) entry
any parameter `--parameter`		`gecko_params: --parameter ...`
any parameter `--parameter <value>`		`gecko_params: --parameter <value> ...`

7 ANGES: Ancestral genome reconstruction

In this last step of the pipeline, ANGESpy3 (a port of ANGES to Python 3) was used with default parameters. In the main experiments made for this work we provided to ANGESpy3 syntenic block scores calculated by the average local DCJ similarity between the block occurrence in the reference genome (grape) and all block occurrences in other species. Besides, the heuristic algorithm was used to reconstruct the PQ-tree. ANGESpy3 can be downloaded from `https://gitlab.ub.uni-bielefeld.de/gi/angespy3`.

Table 6 shows how to configure ANGESpy3 behavior in the `config.yaml` file of our workflow.

Table 6: Mapping of parameters for ancestral reconstruction.

ANGESpy3 behavior	→	Workflow configuration (`config.yaml`) entry
Use local DCJ similarity scores for blocks		`anges_use_sim_weight: True`
Compute scores for blocks (no local DCJ sim. score)		`anges_use_sim_weight: False`
Use heuristics + branch-and-bound		`anges_run_bab: True`
Use heuristics only		`anges_run_bab: False`

8 Calculation of proportions of shared content between genomes

In this work, the proportions of genomic markers attributed to each ancestral chromosome were compared to the proportions derived from Badouin et al.'s [1] gene-based reconstruction. Figure 2 shows the comparison of ancestral genome content w.r.t. coffee and grape chromosomes of this analysis. The genome architecture of grape is closest to the post-γ ancestor, therefore the layout of the grape genome serves as proxy for reconstructing the genome of the post-γ ancestor in this work.

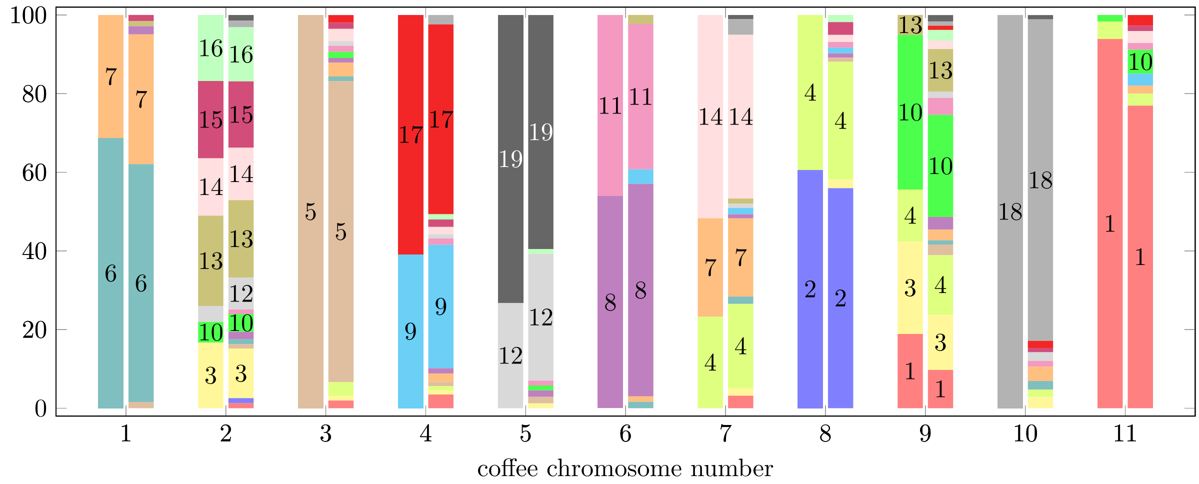

Figure 2: Shared content between coffee and grape genomes in the reconstructed ancestor. For each coffee chromosome (x-axis), each pair of bars shows the proportion shared with grape chromosomes (indicated by the color and chromosome number inside each bar segment) by the ancestral genome of Badouin *et al.* [1] (left) and ours (right), respectively. For better visualization, proportions of ancestral genome contents below 1% are not shown.

The method for calculating such proportions of shared content between the grape genome and one of the other genomes in the reconstructed ancestor is as follows: Given a chromosome C of the other species, let S be the set of syntenic blocks occurring in C that are part of some *contiguous ancestral region*. We compute the percentage of blocks in S that also occur in each of the chromosomes in grape genome. As an example, for the chromosome 1 of coffee genome, the total number of syntenic blocks that are part of some CAR is 385 and, from this total, 233 blocks (60.52%) occur in chromosome 6 and 127 (32.99%) in chromosome 7 of grape genome. Figure 2 shows these proportions.

Based on these calculated proportions, the difference of the layouts given by our ancestral reconstruction and the ancestral reconstruction by Badouin et al. (as computed in the main mansucript) is used as a measure of how much the two reconstructions differ. Our measure is simply the average absolute differences over all chromosomes between our calculated proportions and those reported by Badouin et al.

References

[1] H. Badouin, J. Gouzy, C. J. Grassa, F. Murat, S. E. Staton, L. Cottret, C. Lelandais-Brière, G. L. Owens, S. Carrère, B. Mayjonade, L. Legrand, N. Gill, N. C. Kane, J. E. Bowers, S. Hubner, A. Bellec, A. Bérard, H. Bergès, N. Blanchet, M.-C. Boniface, D. Brunel, O. Catrice, N. Chaidir, C. Claudel, C. Donnadieu, T. Faraut, G. Fievet, N. Helmstetter, M. King, S. J. Knapp, Z. Lai, M.-C. Le Paslier, Y. Lippi, L. Lorenzon, J. R. Mandel, G. Marage, G. Marchand, E. Marquand, E. Bret-Mestries, E. Morien, S. Nambeesan, T. Nguyen, P. Pegot-Espagnet, N. Pouilly, F. Raftis, E. Sallet, T. Schiex, J. Thomas, C. Vandecasteele, D. Varès, F. Vear, S. Vautrin, M. Crespi, B. Mangin, J. M. Burke, J. Salse, S. Muños, P. Vincourt, L. H. Rieseberg, and N. B. Langlade. The sunflower genome provides insights into oil metabolism, flowering and Asterid evolution. *Nature*, 546(7656):148–52, Jun 2017.

[2] M. Visnovská, T. Vinař, and B. Brejová. DNA sequence segmentation based on local similarity. In *Proc. of ITAT*, pages 36–43, 2013.
